# Phospholipase Cε, an Effector of Ras and Rap Small GTPases, Is Required for Airway Inflammatory Response in a Mouse Model of Bronchial Asthma

**DOI:** 10.1371/journal.pone.0108373

**Published:** 2014-09-30

**Authors:** Tatsuya Nagano, Hironori Edamatsu, Kazuyuki Kobayashi, Nobuyuki Takenaka, Masatsugu Yamamoto, Naoto Sasaki, Yoshihiro Nishimura, Tohru Kataoka

**Affiliations:** 1 Division of Molecular Biology, Department of Biochemistry and Molecular and Biology, Kobe University Graduate School of Medicine, Kobe, Japan; 2 Division of Respiratory Medicine, Department of Internal Medicine, Kobe University Graduate School of Medicine, Kobe, Japan; 3 Division of Cardiovascular Medicine, Department of Internal Medicine, Kobe University Graduate School of Medicine, Kobe, Japan; BloodCenter of Wisconsin, United States of America

## Abstract

**Background:**

Phospholipase Cε (PLCε) is an effector of Ras and Rap small GTPases and expressed in non-immune cells. It is well established that PLCε plays an important role in skin inflammation, such as that elicited by phorbol ester painting or ultraviolet irradiation and contact dermatitis that is mediated by T helper (Th) 1 cells, through upregulating inflammatory cytokine production by keratinocytes and dermal fibroblasts. However, little is known about whether PLCε is involved in regulation of inflammation in the respiratory system, such as Th2-cells-mediated allergic asthma.

**Methods:**

We prepared a mouse model of allergic asthma using *PLCε* ^+/+^ mice and *PLCε* ^ΔX/ΔX^ mutant mice in which PLCε was catalytically-inactive. Mice with different *PLCε* genotypes were immunized with ovalbumin (OVA) followed by the challenge with an OVA-containing aerosol to induce asthmatic response, which was assessed by analyzing airway hyper-responsiveness, bronchoalveolar lavage fluids, inflammatory cytokine levels, and OVA-specific immunoglobulin (Ig) levels. Effects of *PLCε* genotype on cytokine production were also examined with primary-cultured bronchial epithelial cells.

**Results:**

After OVA challenge, the OVA-immunized *PLCε* ^ΔX/ΔX^ mice exhibited substantially attenuated airway hyper-responsiveness and broncial inflammation, which were accompanied by reduced Th2 cytokine content in the bronchoalveolar lavage fluids. In contrast, the serum levels of OVA-specific IgGs and IgE were not affected by the *PLCε* genotype, suggesting that sensitization was PLCε-independent. In the challenged mice, *PLCε* deficiency reduced proinflammatory cytokine production in the bronchial epithelial cells. Primary-cultured bronchial epithelial cells prepared from *PLCε* ^ΔX/ΔX^ mice showed attenuated pro-inflammatory cytokine production when stimulated with tumor necrosis factor-α, suggesting that reduced cytokine production in *PLCε* ^ΔX/ΔX^ mice was due to cell-autonomous effect of *PLCε* deficiency.

**Conclusions:**

PLCε plays an important role in the pathogenesis of bronchial asthma through upregulating inflammatory cytokine production by the bronchial epithelial cells.

## Introduction

Allergic asthma is one of the most common chronic inflammatory diseases and is characterized by airway hyper-responsiveness (AHR), accumulation of eosinophils in the airway, increased mucus production by the airway epithelium, increased serum allergen-specific immunoglobulin (Ig)E and IgG levels, *etc.* [1]. In the development of such symptoms, T helper (Th)2 cell subset plays a central role by secreting Th2 signature cytokines, such as interleukin (IL)-4, which promotes IgE class switching in B lymphocytes and Th2 cell survival, IL-5, which is crucial for eosinophil survival thereby contributing to the development of eosinophilic inflammation, and IL-13, which plays a role in differentiation of mast cells and mucus-producing goblet cells [1], [2].

Phospholipase C (PLC) plays a pivotal role in regulation of intracellular signaling pathways by hydrolyzing phosphatidylinositol 4,5-bisphosphate into diacylglycerol and inositol 1,4,5-trisphosphate, which regulate a variety of diacylglycerol target proteins exemplified by protein kinase C isoforms and the intracellular Ca^2+^ ion levels, respectively [3]. In mammals, at least 13 PLC isoforms have been identified and classified into 6 classes (β, γ, δ, ε, ζ, and η) based on the similarities in their structures as well as their regulatory mechanisms [3].

PLCε was first identified as a direct effector of the small GTPase Ras and subsequently shown to be activated by the small GTPases Rap1 and RhoA, and α_12_ and β_1_γ_2_ subunits of heterotrimeric G protein [4]–[7]. Extracellular ligands, such as epidermal growth factor [8], platelet-derived growth factor [9], lysophosphatidic acid [10], and sphingosine-1-phosphate [10] can induce the activation of PLCε through upregulating the above-mentioned small GTPases and G proteins. In mammals, PLCε is expressed in non-immune cells such as epidermal keratinocytes, dermal fibroblasts, and epithelial cells, but not in immune cells such as lymphocytes, granulocytes, macrophages, and dendritic cells [11], [12].

One of the physiological roles of PLCε is the augmentation of inflammation through upregulation of pro-inflammatory cytokine production by non-immune cells [11]–[15]. We have reported that *PLCε* ^ΔX/ΔX^ mice, which are homozygous for the allele devoid of the lipase activity of PLCε [16], exhibited attenuated skin inflammation induced by phorbol-12-myristate-13-acetate painting [11] or ultraviolet B (UVB) irradiation [13]. We and others have shown that knockout or knockdown of *PLCε* in cultured cells inhibited production of proinflammatory molecules induced by stimulation with ligands such as tumor necrosis factor (TNF)- α [14], lysophosphatidic acid [10], and sphingosine-1-phosphate [10], or with UVB irradiation [13]. In a Th1 cell-mediated allergic contact hypersensitivity murine model, PLCε plays a crucial role by mediating proinflammatory molecule expression in the non-immune skin cells in response to Th1 cell infiltration [12]. However, the role of PLCε in Th2 cell-mediated inflammation remains to be clarified. In this study, we aim to determine the role of PLCε in bronchial asthma.

## Methods

### Animals

Mice with the inactivated *PLCε* allele (*PLCε* ^ΔX^), the allele devoid of the lipase activity of PLCε by an in-frame deletion at the catalytic X domain [16], had been backcrossed to C57BL/6JJcl mice (CLEA Japan, Inc., Tokyo, Japan) for at least 8 generations and were maintained at the specific pathogen-free animal facility in Kobe University Graduate School of Medicine. The use and care of the animals were reviewed and approved by the Institutional Animal Care and Use Committee of Kobe University (Permit Numbers: P100616, P100617, P130401, and P130403).

### Chemicals and antibodies

Recombinant murine TNF-α (315-01, PeproTech, Rocky Hill, NJ) was used. IKK inhibitor III (also known as BMS-345541; 401480, Calbiochem) and U73122 (662035, Calbiochem) were purchased. Anti-PLCε antibody against the C-terminal peptide of mouse PLCε was in-house generated [17] and capable of reacting with the lipase-dead protein present in PLCε mice [16]. Other antibodies used are as follows: Anti-CC chemokine ligand (Ccl)2 (sc-1785, Santa Cruz), anti-chemokine (C-X-C motif) ligand (Cxcl)2 (AF-452-NA, R and D Systems, Minneapolis, MN), PerCP-Cy5.5-conjugated anti-CD45R (RA3-6B2, eBioscience), APC/Cy7-conjugated anti-CD3ε (145-2C11, BioLegend, San Diego, CA), PE-conjugated anti-CD4 (RM4-5, BD Biosciences, San Jose, CA), APC-conjugated anti-CD11c (N418, eBioscience), FITC-conjugated anti-MHC class II (M5/114.15.2, eBioscience), and anti-pan cytokeratin (C-11, abcam). CF dye-labeled secondary antibodies were purchased from Biotium (Hayward, CA).

### Induction of general anesthesia for operations of mice

To induce general anesthesia, mice were given dexmedetomidine (0.3 mg/kg; Maruishi Pharmaceutical, Osaka, Japan), midazolam (4 mg/kg; Astellas Pharma, Tokyo, Japan) and butorphanol tartrate (5 mg/kg; Meiji Seika Pharma, Tokyo, Japan) by intraperitoneal injection.

### Induction of bronchial asthma

Mice at 6 weeks of age were sensitized by intraperitoneal injections of 500 µl of phosphate-buffered saline (PBS) containing 10 µg of ovalbumin (OVA; Sigma-Aldrich, St. Louis, MO) complexed with 1 mg aluminum hydroxide (Sigma-Aldrich) at days 0, 7 and 14. Sham immunization was performed by intraperitoneal injections of PBS alone. At days 21, 22 and 23, these sensitized mice were challenged for 30 min with PBS aerosol with or without 1% (w/v) OVA using an ultrasonic nebulizer (NE-U17; Omron, Kyoto, Japan).

### Preparation and staining of histologic specimens

The lungs were perfused with PBS followed by fixation by intratracheal instillation of phosphate-buffered paraformaldehyde or OCT compound (Sakura Finetek, Tokyo, Japan) for embedding in paraffin or OCT compound, respectively. They were sectioned and subjected to hematoxylin and eosin (H&E) staining, periodic acid-Schiff (PAS) staining and immunostaining as described [18], [19].

### Airway responsiveness

Twenty-four hours after the last aerosol challenge, response to methacholine was measured by an invasive approach [20]. Briefly, generally-anesthetized mice were surgically intubated and connected to the plethysmograph chambers with the ventilation system (Elan RC system, Buxco, Wilmington, NC). Airway resistance was recorded for 3 min after each aerosol challenge and analyzed by Buxco BioSystem XA software. Data are expressed as an increase over the baseline set at 1 cm H_2_O/ml/s.

### Analysis of bronchoalveolar lavage fluid (BALF)

The lungs were lavaged through a tracheal cannula with 0.8 ml of PBS three times at 24 h after the last aerosol challenge. The BALF was collected and centrifuged at 1,500×rpm for 5 min at 4°C to pellet leukocytes, whose number was determined by using hemocytometer. The leukocytes were then subjected to cytospin preparation and stained with Diff-Quick (Sysmex, Kobe, Japan). At least 200 leukocytes on each slide were subjected to differential counting of macrophages, neutrophils, lymphocytes and eosinophils according to the standard morphological criteria. Concentrations of IL-4, IL-5, IL-13 and IFN-γ in the BALF were determined with the ELISA kits, BMS613, BMS610, BMS6015 and BMS606, respectively (eBioscience, San Diego, CA).

### Enzyme-linked immunosorbent assay (ELISA) for serum OVA-specific Ig

The serum Ig levels were determined by using DS mouse IgE ELISA OVA kit (FCMA007A, DS Pharma Biochemical, Osaka, Japan) for OVA-specific IgE or by using the sandwich ELISA method with ELISA starter accessory kit (E101, Bethyl Laboratories, Montgomery, TX) along with horseradish peroxidase-conjugated goat anti-mouse IgG1 and IgG2a (E90-105 and E90-107, respectively, Bethyl Laboratories). All assays were performed in duplicate of each sample.

### OVA transport to the thoracic lymph nodes

A total of 50 µl of 10 mg/ml fluorescein-conjugated OVA (023020, Life Technologies, Carlsbad, CA) were intratracheally instilled into the sensitized mice at day 21. Twenty-four hours later, the location of OVA was immunohistologically analyzed.

### Thoracic lymph node leukocyte analysis

All visible thoracic lymph nodes were collected from the sensitized mice 1 day after the last challenge with vehicle alone or OVA, and subjected to isolation of leukocytes. Leukocytes were stained for cell surface markers for flowcytometric analysis using Attune Acoustic Focusing Cytometer (Life Technologies), where at least 20,000 events were collected in each analysis. Data were further analyzed by FlowJo 8 software (Tree Star, Ashland, OR).

### Reverse transcription (RT)-polymerase chain reaction (PCR)

Total cellular RNA preparation, RT-PCR and quantitative RT-PCR (qRT-PCR) were performed essentially as described [21]. Relative mRNA levels were calculated with the ΔΔCt method using the *β-actin* mRNA as an internal control. Primers used in this study are as follows: 5′-aacgccaactgggcacctc-3′ and 5′-ctgaggccagccaggaactc-3′ for *PLCε,* 5′-atgaagatcaagatcattgctcctc-3′ and 5′-acatctgctggaaggtggacag-3′ for *β-actin* [21], 5′-ttgtcaccaagctcaagagaga-3′ and 5′-gaggtggttgtggaaaaggtag-3′ for *Ccl2* [22], 5′-cccatccctgggaacatcgtg-3′ and 5′-cacagggctccttctggtgctg-3′ for *Ccl19* [23], 5′-agtttgccttgaccctgaag-3′ and 5′-ctttggttcttccgttgagg-3′ for *Cxcl2* [19], 5′-atcccggcaatcctgttctt-3′ and 5′-agttctcttgcagcccttgg-3′ for *Ccl21* [23], 5′-aggctaccctgaaactgag-3′ and 5′-ggagattgcatgaaggaatacc-3′ for *thymic stromal lymphopoietin* (*Tslp*) [24], 5′-cagatggcttcccctgtgttct-3′ and 5′-aaggtgagtccttggcgtgaac-3′ for *chemokine (C-X3-C motif) ligand* (*Cx3cl*)*1* [23], 5′-gagccaacgtcaagcatctg-3′ and 5′-cgggtcaatgcacacttgtc-3′ for *Cxcl12* [25], 5′-tctgctgcctgtcacatcatc-3′ and 5′-ggacattgaattcttcactgatattca-3′ for *IL-7* [26], 5′-ggcarcagagacaccaattacct-3′ and 5′-tggcattggtcagctgtaaca-3′ for *IL-9* [27], 5′-cacactgcgtcagcctacaga-3′ and 5′-tgtggtaaagtgggacggagtt-3′ for *IL-25* [28], 5′-cctccctgagtacatacaatgacc-3′ and 5′-gtagtagcacctggtcttgctctt-3′ for *IL-33* [29].

### Bronchial epithelial cell cultures

Primary culture of bronchial epithelial cells was prepared from adult naïve mice as described [30]. The purity of the culture was over 99% as assessed by immunostaining for the epithelial cell marker, cytokeratin (data not shown) [31].

### Statistics and data reproducibility

Data are expressed as the mean ± SD. If p value obtained by unpaired two-tailed Student’s t-test was smaller than 0.05, difference was considered to be statistically significant.

## Results

### Alleviation of asthma symptoms in *PLCε* ^ΔX/ΔX^ mice

**Figure 1:**
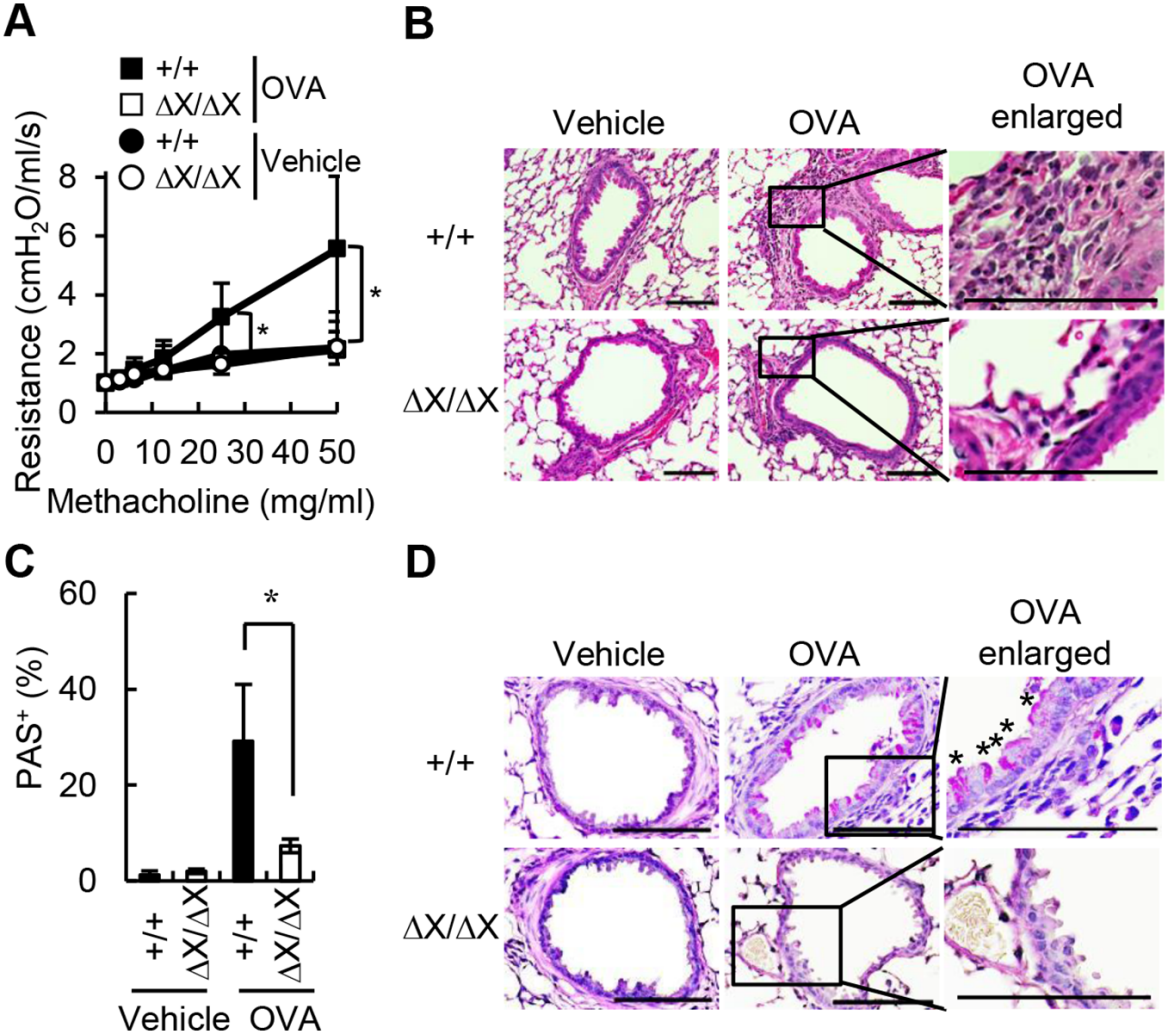
Attenuated asthmatic response in *PLCε* ^ΔX/ΔX^ mice. (A) AHR to methacholine. AHR was assessed in OVA-sensitized *PLCε* ^+/+^ (*filled symbols*) and *PLCε* ^ΔX/ΔX^ (*open symbols*) mice 1 day after the last challenge with the aerosol containing OVA (*squares*) or with vehicle alone (*circles*). Resistance is expressed as an increase over the baseline set at 1 cmH_2_O/ml/s. Data are shown as the mean ± SD obtained with 3 or 4 mice of each group. *, p<0.05 between the OVA-challenged *PLCε* ^+/+^ and *PLCε* ^ΔX/ΔX^ mice. (B) H&E staining of airway sections. Airway sections were prepared from the OVA-sensitized mice of the indicated *PLCε* genotype 1 day after the last challenge either with OVA-containing aerosol or with vehicle alone as indicated. *OVA enlarged* show the enlargement of the boxed areas in *OVA*. *Bars,* 100 µm. (C, D) Frequency of PAS^+^ cells. Airway sections prepared as in (B) were subjected to PAS staining to vitalize mucus-producing cells. Nuclei were counterstained with hematoxylin. Representative sections are shown in (D), where *OVA enlarged* show the enlargement of the boxed areas in *OVA* and asterisks denote PAS^+^ cells. PAS^+^ bronchial epithelial cells and total epithelial cells were counted on the specimens prepared from 3 or 5 mice of each group, and the percentage of PAS^+^ epithelial cells was determined as 100×(PAS^+^ cell number)/(total epithelial cell number) (%). Data in (C) are expressed as the mean ± SD. *, p<0.05 between the OVA-challenged two *PLCε* genotypes. *Bars in* (D), 100 µm.

As multiple tissue Northern blot analyses indicated the presence of the *PLCε* mRNA in the lung [4], [5], we asked whether PLCε played a role in the development of bronchial asthma by using a mouse model of OVA-induced allergic bronchial asthma. To induce asthma, *PLCε* ^+/+^ and *PLCε* ^ΔX/ΔX^ mice were sensitized with OVA and subsequently challenged with an aerosol of PBS alone or that containing OVA for three consecutive days. Twenty-four hours after the last aerosol challenge, we performed a methacholine challenge test for examination of the AHR to muscarinic cholinergic stimulation (Figure 1A). As expected, *PLCε* ^+/+^ mice challenged with OVA exhibited AHR in a methacholine dosage dependent-manner. As compared to *PLCε* ^+/+^ mice, *PLCε* ^ΔX/ΔX^ mice challenged with OVA showed substantially attenuated AHR. To gain further insights, we performed histological analyses 24 h after the last aerosol challenge of the sensitized mice (Figure 1B). In *PLCε* ^+/+^ mice challenged with OVA, a large number of inflammatory cells were accumulated around the bronchus. In contrast, such inflammatory cell accumulation induced after OVA challenge was blunted in *PLCε* ^ΔX/ΔX^ mice, indicating attenuated airway inflammation in *PLCε* ^ΔX/ΔX^ mice. On the same specimens, we also carried out PAS staining to visualize mucin-producing goblet cells (Figure 1C and D). The staining indicated that the frequency of PAS^+^ goblet cells in the OVA-challenged *PLCε* ^ΔX/ΔX^ mice was smaller than that in the OVA-challenged *PLCε* ^+/+^ mice, indicating reduced mucus production in the airway of *PLCε* ^ΔX/ΔX^ mice. These results taken together indicated that *PLCε* deficiency relieved asthma symptoms and suggested that *PLCε* had a role in the asthmatic phenotype development.

### Attenuation of Th2 cell-mediated inflammation in the lung of *PLCε* ^ΔX/ΔX^ mice

**Figure 2:**
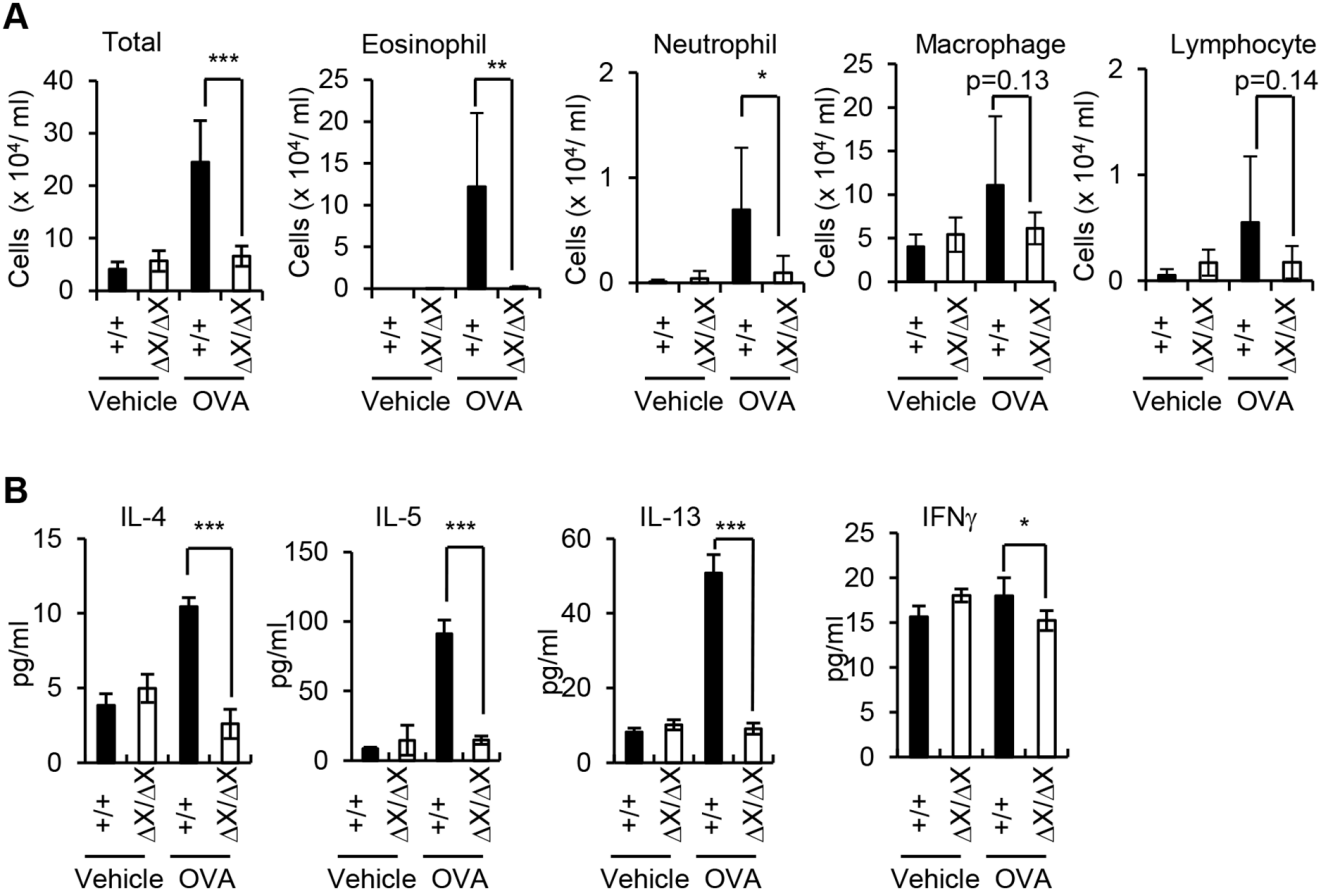
Reduced Th2 response in the respiratory system of *PLCε* ^ΔX/ΔX^ mice. (A) Total and differential leukocyte counts. BALF was collected 1 day after the last challenge of the OVA-sensitized *PLCε* ^+/+^ (*filled bars*) and *PLCε* ^ΔX/ΔX^ (*open bars*) mice with the aerosol containing OVA or with vehicle alone as indicated. For differential leukocyte counting, leukocytes were pelleted from the collected BALF and stained with Diff-Quick. Data are expressed as the mean ± SD obtained with 6 to 10 mice of each group. *, p<0.05; **, p<0.01; ***, p<0.001; *n.s.*, statistically not significant. (B) Cytokine content in BALF. The supernatant of the centrifugation obtained in *A* was subjected to the determination of the BALF cytokine levels by ELISA. Data are expressed as the mean ± SD. *, p<0.05; ***, p<0.001.

To identify the role of PLCε in the development of asthma phenotypes, we examined the effects of the *PLCε* genotype on leukocyte infiltration associated with asthmatic inflammation. To this end, BALF was collected 24 h after the last aerosol challenge from the OVA-sensitized mice and subjected to differential leukocyte counting by staining with Diff-Quick (Figure 2A and S1 in File S1). In *PLCε* ^+/+^ mice, OVA challenge resulted in a great increase in the number of leukocytes, most of which were identified as eosinophils. In contrast, in *PLCε* ^ΔX/ΔX^ mice, the increase of leukocytes, particularly eosinophils and neutrophils, was indeed suppressed. A similar trend was seen in the number of lymphocytes and macrophages but the difference associated with the *PLCε* genotype was statistically insignificant (p>0.05). These results indicated that the *PLCε* genotype affected the development of eosinophilia in the airway.

We next assessed the cytokine concentrations in the collected BALF (Figure 2B). As determined by ELISA, the BALF prepared from the OVA-challenged *PLCε* ^ΔX/ΔX^ mice contained much less Th2 signature cytokines, IL-4, IL-5, and IL-13, than that from the OVA-challenged *PLCε* ^+/+^ mice. In contrast, the level of one of the Th1 cytokines, IFNγ, was not increased very much even in *PLCε* ^+/+^ mice.

**Figure 3:**
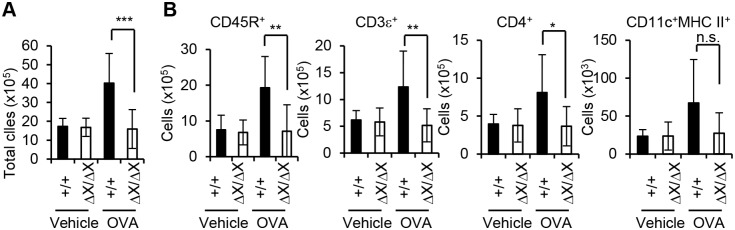
Reduced number of Th2 cells in the thoracic lymph nodes of *PLCε* ^ΔX/ΔX^ mice. (A) Total leukocyte count of the thoracic lymph nodes. All visible thoracic lymph nodes were collected from the sensitized mice with the indicated *PLCε* genotype 1 day after the last challenge with vehicle alone or OVA (6 to 15 mice of each group), and subjected to isolation of leukocytes. Leukocyte number was determined using a hematocytometer. Data are expressed as the mean ± SD. ***, p<0.001. (B) Flowcytometric analysis of leukocytes. Collected leukocytes in (A) were further analyzed flowcytometrically for the expression of the indicated cell surface antigens. Data are expressed as the mean ± SD. *, p<0.05; **, p<0.01. *n.s.*, statistically not significant.

We also analyzed leukocytes in the regional lymph nodes 24 h after the last aerosol challenge (Figure 3). OVA challenge increased the total leukocyte number in the thoracic lymph nodes of *PLCε* ^+/+^ mice (Figure 3A). Flow cytometric analyses for the surface antigens indicated a substantial increase in the number of CD45R^+^ B lymphocytes, which accounted for about 50% of the thoracic lymph node leukocytes in the OVA-challenged *PLCε* ^+/+^ mice (Figure 3B). In contrast, in *PLCε* ^ΔX/ΔX^ mice, OVA challenge failed to increase total leukocyte count (Figure 3A) as well as B lymphocyte count (Figure 3B). A similar trend was seen in the numbers of CD3ε^+^ T lymphocytes and CD4^+^ leukocytes. On the other hand, no statistical difference depending on the *PLCε* genotype was observed in the number of CD11c^+^MHC II^ +^ dendritic cells. These results indicated that Th2 cell-mediated inflammation was attenuated in the lung of *PLCε* ^ΔX/ΔX^ mice.

### No effect of the *PLCε* genotype on the serum OVA-specific Ig levels in the OVA-sensitized mice

**Figure 4:**
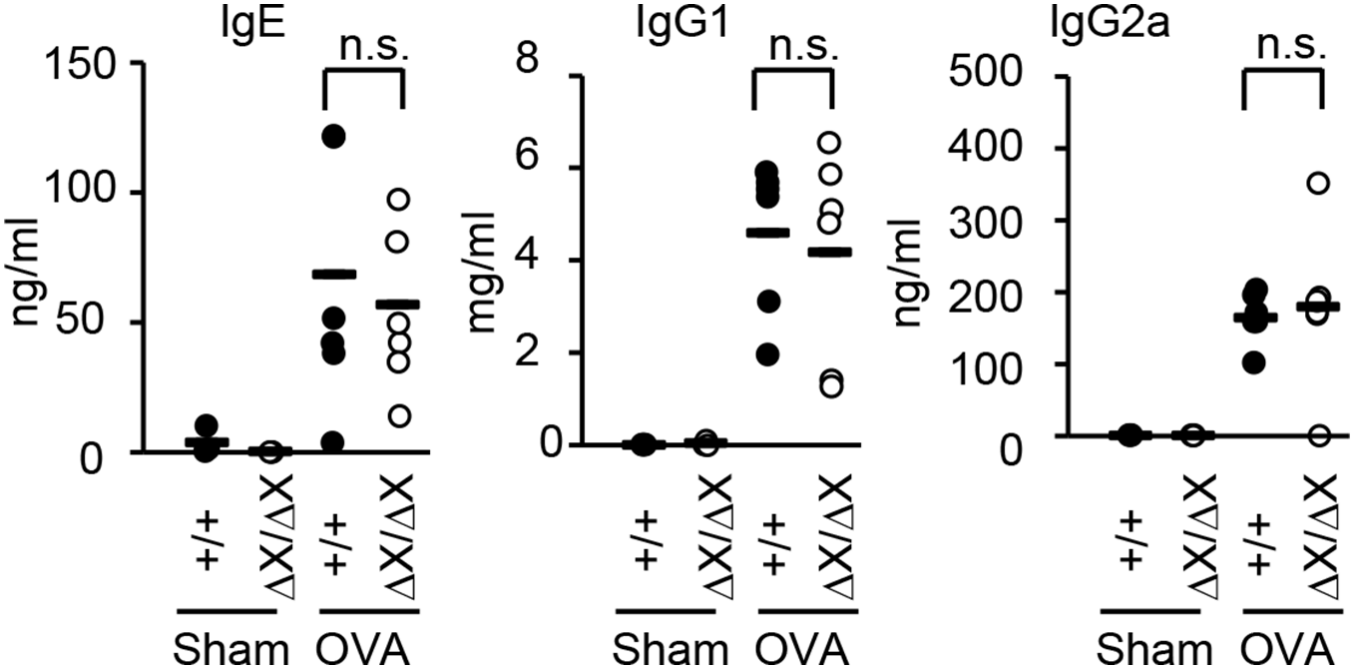
No effect of the *PLCε* genotype on OVA-specific IgE and IgGs. Serums were prepared from the mice with the indicated *PLCε* genotype 7 days after the last injection of OVA (*OVA*) or vehicle alone (*Sham*), and they were subjected to the determination of the indicated Ig levels using ELISA. *Lines* indicate the mean, and each symbol represents an individual mouse of *PLCε* ^+/+^ (*filled symbols*) or *PLCε* ^ΔX/ΔX^ (*open symbols*). *n.s.*, statistically not significant.

We examined the effect of the *PLCε* genotype on the serum levels of OVA-specific IgE and IgGs 7 days after the last intraperitoneal injection of OVA (Figure 4). The levels of OVA-specific IgE, IgG1, and IgG2 were indeed increased after the OVA immunization, but these increases were not affected by the *PLCε* genotype. Thus, PLCε seemed to be dispensable for the sensitization with OVA and to play a role in the elicitation phase.

### No association between the *PLCε* genotype and the efficiency of the allergen transfer by CD11c^+^ dendritic cells

**Figure 5:**
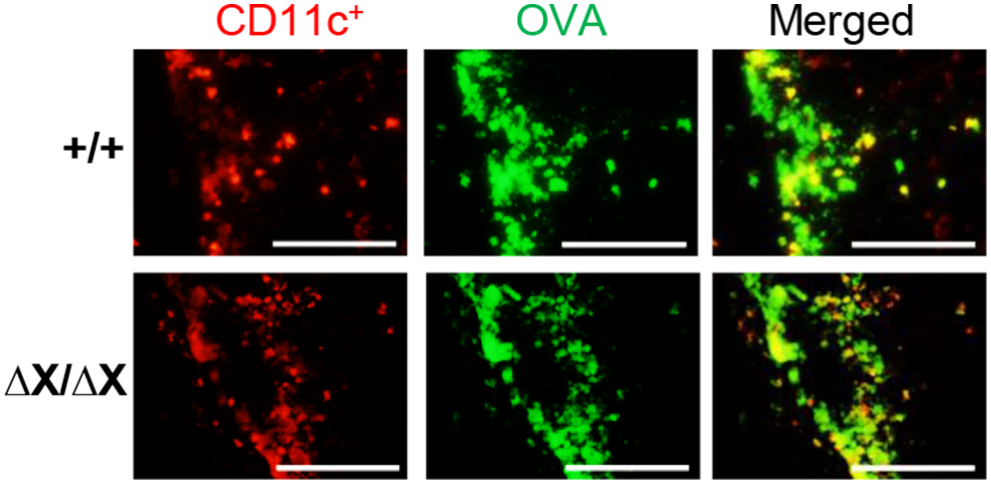
No effect of the *PLCε* genotype on the antigen transport by CD11c^+^ dendritic cells. Fluorescein-conjugated OVA (*green*) was instilled into the OVA-sensitized *PLCε* ^+/+^ (*upper*) and *PLCε* ^ΔX/ΔX^ (*lower*) mice. Twenty-four hours later, the thoracic lymph nodes were sampled for staining for CD11c (*red*) and fluorescence-microscopically observed. Cells doubly positive for fluorescein-conjugated OVA and CD11c (*yellow* in *Merged*) were identified as CD11c^+^ dendritic cells carrying OVA. *Bars,* 100 µm.

Next, we paid attention to CD11c^+^ dendritic cells because they are responsible for the transferring allergens to the regional lymph nodes where Th2 cell polarization was identified upon antigen presentation by dendritic cells [32]. To this end, we studied the effect of the *PLCε* genotype on the ability of CD11c^+^ dendritic cells to transfer OVA to the regional lymph nodes. To visualize OVA, we intratracheally instilled traceable fluorescein-conjugated OVA into the sensitized mice and 24 h later analyzed the thoracic lymph nodes. Fluorescence microscopy demonstrated that there was no apparent difference associated with the *PLCε* genotype in the frequency of CD11c^+^ dendritic cells carrying fluorescein-conjugated OVA (Figure 5), suggesting that CD11c^+^ dendritic cells in *PLCε* ^ΔX/ΔX^ mice were capable of transferring allergens. These results also supported the notion that PLCε was dispensable for sensitization with allergens, which involves the antigen-presenting function of dendritic cells.

### Crucial role of PLCε in cytokine production from non-immune cells during the development of bronchial inflammation

**Figure 6:**
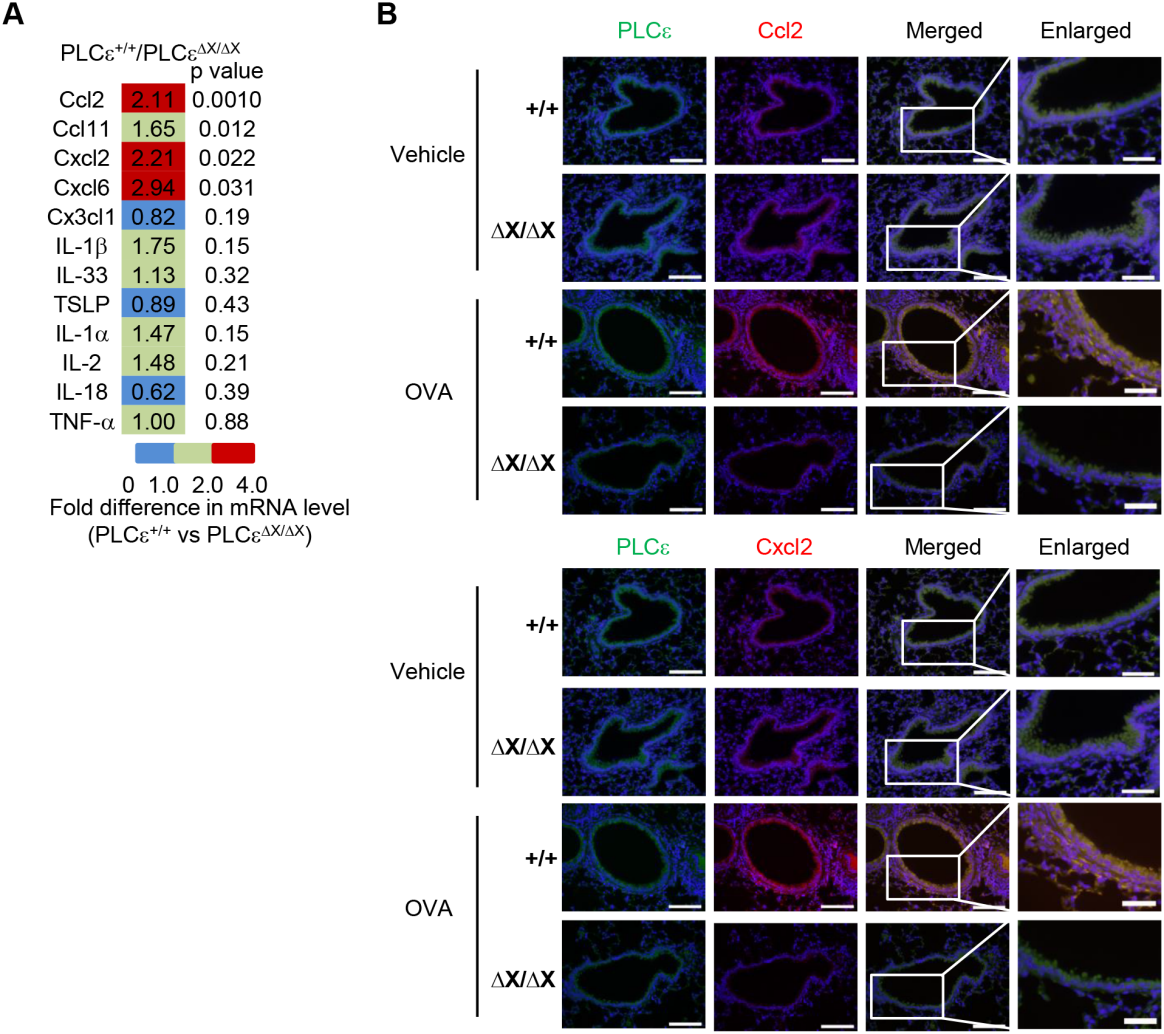
Attenuation of inflammatory cytokine production by *PLCε* deficiency. (A) Comparison of cytokine mRNA levels of the whole lungs of the OVA-challenged *PLCε* ^+/+^ and *PLCε* ^ΔX/ΔX^ mice. OVA-sensitized mice were challenged with OVA, and 1 day later, their whole lungs were collected for RNA preparation. RNA was pooled from the lungs of 6 animals of each group and subjected to qRT-PCR to determine the relative cytokine mRNA levels. Fold difference was obtained by dividing the mRNA level in OVA-challenged *PLCε* ^+/+^ mice with that in OVA-challenged *PLCε* ^ΔX/ΔX^ mice. *p values* shown were derived from the comparisons between the OVA-challenged two *PLCε* genotypes. (B) Immunostaining for cytokines and PLCε. OVA-sensitized mice of the indicated *PLCε* genotype were challenged with vehicle alone or OVA, and 1 day later the airway sections were prepared for immunostaining for Ccl2 (*red* in *upper*) and Cxcl2 (*red* in *lower*) as well as PLCε (*green*). The lipase-dead mutant PLCε in *PLCε* ^ΔX/ΔX^ mice could also be detected by the anti-PLCε antibody against the C-terminus of PLCε. Nuclei were visualized by 4′,6-Diamidino-2-Phenylindole (DAPI) staining (*blue*). *Enlarged* show the enlargement of the boxed areas in *Merged*. *Bars,* 100 µm.

It was reported that attenuation of Th1 cell-mediated allergic contact hypersensitivity in *PLCε* ^ΔX/ΔX^ mice is owing to reduced proinflammatory cytokine production by *PLCε* deficient epidermal keratinocytes and dermal fibroblasts in the elicitation phase [12]. We therefore hypothesized that the attenuation of asthmatic inflammation in the elicitation phase in *PLCε* ^ΔX/ΔX^ mice might be due to reduced cytokine production by the lung structural cells. To test this hypothesis, we examined the effects of the *PLCε* genotype on the expression of cytokine mRNAs in the whole lungs 24 h after the last aerosol challenge of the sensitized mice (Figures 6A and S2 in File S1). As determined by qRT-PCR, OVA challenge markedly increased the expression of an array of proinflammatory cytokines, including *Ccl2* and *Cxcl2* in *PLCε* ^+/+^ mice. In contrast, the OVA-challenged *PLCε* ^ΔX/ΔX^ mice exhibited reduced expression of *Ccl2* and *Cxcl2*. We failed to detect the effect of the *PLCε* genotype on the expression of some cytokines implicated in the pathogenesis of asthma including *IL-33* [33], *tslp* [34], and *Cx3cl1* [35]. Because immunostaining using an antibody against the C-terminus of PLCε [16], [17] demonstrated that PLCε was highly expressed in non-immune structural cells including alveolar epithelial cells, bronchial epithelial cells, and smooth muscle cells (Figure S3 in File S1), it was very likely that PLCε deficiency affected these structural cells leading to reduced pro-inflammatory cytokine production upon OVA challenge. Indeed, multiple immunostaining demonstrated that cells positive for wild-type PLCε, particularly bronchial epithelial cells, abundantly expressed both Ccl2 and Cxcl2 upon OVA challenge and that bronchial epithelial cells in *PLCε* ^ΔX/ΔX^ mice were negative for expression of these chemokines even after OVA challenge (Figure 6B).

### Role of PLCε in TNF-α-induced cytokine gene expression in cultured bronchial epithelial cells

**Figure 7:**
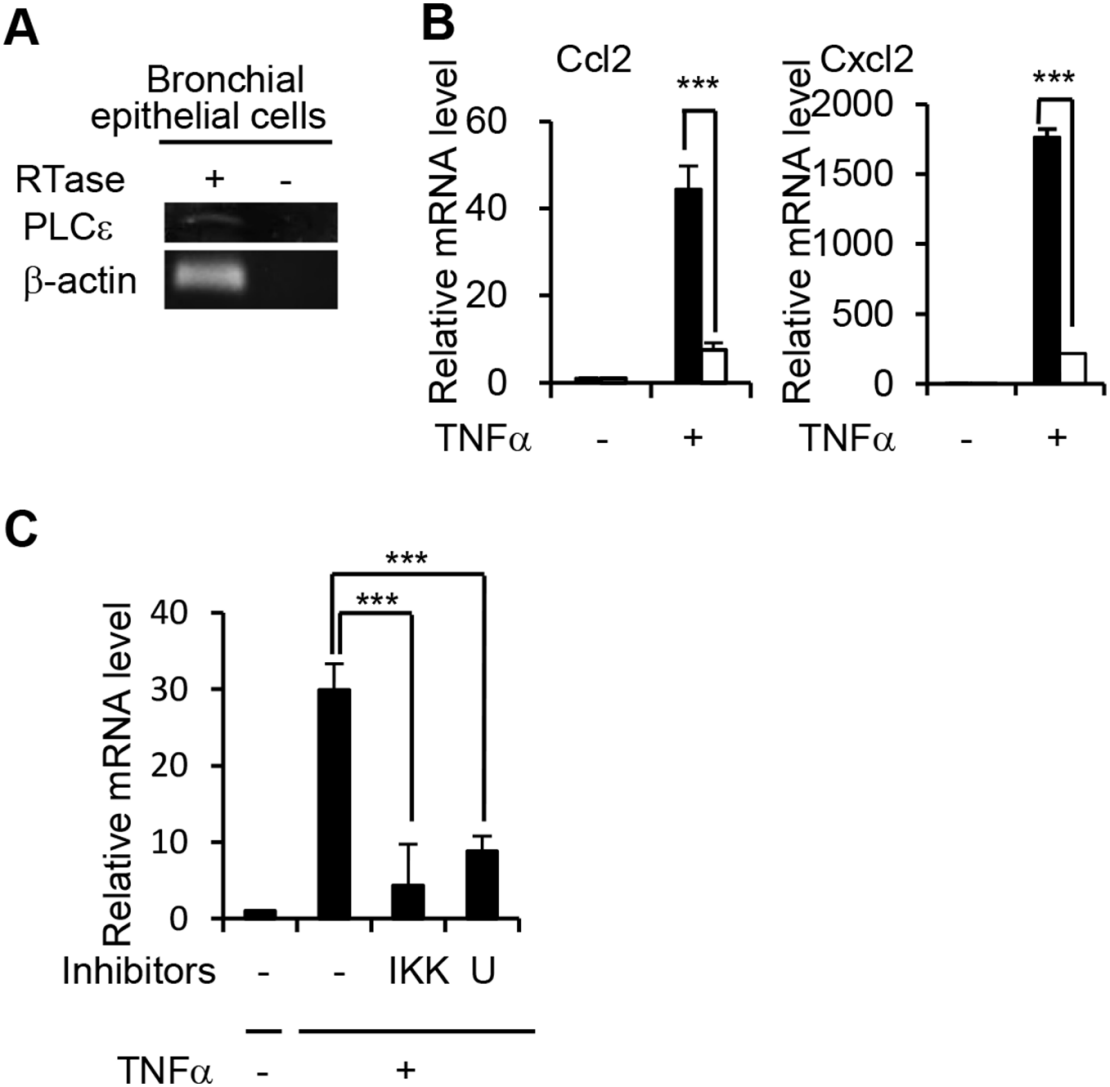
Inhibition of TNF-α-induced cytokine gene activation by *PLCε* deficiency in primary-cultured bronchial epithelial cells. (A) Assessment of *PLCε* expression in primary-cultured bronchial epithelial cells was by RT-PCR. Primary cultures of bronchial epithelial cells were prepared from naïve adult *PLCε* ^+/+^ mice. RNA was prepared for the first-strand preparation with (*+*) or without (*−*) reverse transcriptase (*RTase*) as indicated. (B) Effects of the *PLCε* genotype on *Ccl2* and *Cxcl2* expression induced by TNF-α. Primary cultures of bronchial epithelial cells prepared from naïve adult *PLCε* ^+/+^ (*filled bars*) and *PLCε* ^ΔX/ΔX^ (*open bars*) mice were treated without (*−*) or with (+) 10 ng/ml TNF-α for 3 h. RNA was purified and subjected to qRT-PCR to determine the mRNA levels of *Ccl2* and *Cxcl2*. Data are representative of three independent experiments and expressed as the mean ± SD obtained by triplicate determinations. *, p<0.05; **, p<0.01; ***, p<0.001 between *PLCε* ^+/+^ (*filled bars*) and *PLCε* ^ΔX/ΔX^ (*open bars*) cells stimulated with TNF-α. (C) Effects of intracellular signaling inhibitors on *Ccl2* expression induced by TNF-α stimulation. Primary-cultured *PLCε* ^+/+^ bronchial epithelial cells prepared as in (B) were pretreated with dimethyl sulfoxide vehicle alone (*−*), 3 µM IKK inhibitor III (*IKK*), or 5 µM U73122 (*U*) for 10 min. Subsequently, they were stimulated with 10 ng/ml TNF-α for 3 h. QRT-PCR was carried out to determine the *Ccl2* mRNA level, and data are presented as in (B). ***, p<0.001; *n.s.*, statistically not significant.

The data presented so far suggested that PLCε is involved in the upregulation of proinflammatory cytokine production in bronchial epithelial cells of the sensitized mice after OVA challenge. To gain insights into the mechanism of PLCε-dependent expression of proinflammatory cytokines, we performed *in vitro* studies using primary-cultured bronchial epithelial cells, where the presence of the *PLCε* mRNA was confirmed by RT-PCR (Figure 7), prepared from naïve mice with the two different *PLCε* genotypes. When stimulated with TNF-α, a cytokine which was reported to efficiently activate these cytokine genes [14], [36], cells prepared from *PLCε* ^ΔX/ΔX^ mice exhibited reduced expression of the *Ccl2* and *Cxcl2* mRNAs compared to those from *PLCε* ^+/+^ mice (Figure 7B), suggesting a role of PLCε in activation of proinflammatory cytokine genes. The mechanism whereby TNF-α induced activation of PLCε is presently unclear. TNF-α may indirectly activate PLCε through humoral factor(s) secreted from the TNF-α-stimulated bronchial epithelial cells as our previous studies with skin-derived cells suggested the requirement of such secondary factors for the *PLCε* genotype-dependent cellular responses to TNF-α [12]. Our previous results obtained with cultured human keratinocytes indicated that TNF-α-induced expression of *Ccl2* requires not only PLCε but also nuclear factor-κB (NF-κB) activity [14]. We therefore asked whether IκB kinase (IKK), an upstream kinase capable of stimulating NF-κB-mediated transcription, was required for induction of *Ccl2* mRNA in TNF-α-activated bronchial epithelial cells. Pretreatment with the IKK inhibitor (IKK inhibitor III) significantly blocked the TNF-α-induced *Ccl2* mRNA induction (Figure 7C). In addition, the pan-PLC inhibitor (U73122) reduced the *Ccl2* mRNA in TNF-α-activated cells (Figure 7C), being consistent with the data obtained with *PLCε* ^ΔX/ΔX^ cells (Figure 7B). These results suggested a crucial role of the IKK-NF-κB pathway and that of the lipase activity of PLCε in TNF-α-induced expression of inflammatory cytokines including Ccl2 in bronchial epithelial cells.

## Discussion

We have shown here that PLCε plays a crucial role in the pathogenesis of a mouse model of bronchial asthma. Pathologic analyses indicated that *PLCε* deficiency inhibits Th2 cell-mediated responses, which include eosinophilia and elevated production of Th2 signature cytokines such as IL-4, IL-5, and IL-13 (Figure 2), suggesting that PLCε plays a crucial role in regulation of Th2 cell-mediated inflammation although its significance in other pathological conditions of Th2 cell-mediated immunity remains to be clarified. Intriguingly, *PLCε* deficiency effectively compromised the production of an array of proinflammatory cytokines exemplified by Ccl2 and Cxcl2 upon OVA challenge (Figure 6) without affecting the serum levels of OVA-specific IgGs and IgE after OVA immunization (Figure 4). These results indicate that PLCε plays a crucial role in the elicitation phase but not the sensitization phase.

As shown by immunostaining analyses (Figure 6B), bronchial epithelial cells were highly positive for PLCε and they produced Ccl2 and Cxcl2 in response to OVA challenge. This OVA-challenge-induced cytokine production was highly associated with the *PLCε* genotype. Because both Ccl2 and Cxcl2 were reported to have a role in induction of AHR and chemoattraction of leukocyte in a mouse model of OVA-induced asthma [37]–[39], reduced production of these cytokines in bronchial epithelial cells may contribute to the alleviation of asthmatic phenotypes observed in *PLCε* ^ΔX/ΔX^ mice. In Th2-cell-mediated allergic asthma, many types of cells, which include not only bronchial epithelial cells but also leukocytes accumulated at the site of inflammation and other structural cells like fibroblasts, produce a variety of pro-inflammatory molecules. To further clarify the role of bronchial epithelial cells whose cytokine production is affected by the PLCε-mediated signaling activity, epithelial-cell-specific inactivation of PLCε, such as that achieved by tissue-specific knock out of the *PLCε* gene in mice, should be carried out in future studies. We failed to detect the effects of the *PLCε* genotype on expression of *IL-33*, *tslp*, and *Cx3cl1*, which are also implicated in the pathogenesis of asthma [26]–[28], suggesting that the extent of the contribution of PLCε to the expression levels may differ depending on the nature of inflammatory molecules.

Our *in vitro* studies using primary cultures of bronchial epithelial cells suggested that PLCε is required for the TNF-α-induced activation of the *Ccl2* and *Cxcl2* genes (Figure 7). In addition, the data obtained with IKK inhibitor III suggested that IKK-stimulated transcriptional activity of NF-κB is required for activation of these cytokine genes (Figure 7C), being consistent with the previously reported results derived from the analysis of the promoter region of these cytokine genes [40], [41]. A recent study using astrocyte primary culture suggested the possible role of NF-κB in PLCε-mediated activation of proinflammatory genes upon stimulation of G-protein-coupled receptors [10]. However, it seems unlikely that TNF-α receptor activation is directly linked to PLCε activation in bronchial epithelial cells because studies using primary-cultured keratinocytes and dermal fibroblasts suggested that TNF-α-induced activation of PLCε is mediated by humoral factor(s) that might be secreted by TNF-α-activated cells [12]. Further studies are needed to clarify the nature of the PLCε-mediated signaling pathway leading to the cytokine induction.

Our present study may give a clue to a novel strategy, *i. e.* inhibition of the PLCε activity, for the care of bronchial asthma patients; small-molecule PLCε inhibitors may effectively alleviate asthma phenotypes in human patients. Because blockade of other PLC isoforms such as PLCδ1 has been shown to promote inflammation in animal models [42], [43] and currently-available PLC inhibitors including U73122 totally lack the isoform specificity [44], therapeutic use of this strategy will need the development of a new class of compounds with specific inhibitory activity on PLCε.

## Supporting Information

File S1 **Figure S1. ELISA of plasma histamine levels.** Plasma samples were collected from the sensitized PLCε^+/+^ (*closed bars*) and PLCε^−/−^ (*open bars*) mice 24 h after the last challenge with vehicle alone or OVA. Data are expressed as the mean ± SD obtained with 3 mice of each group. **Figure S2. Effects of the PLCε genotype on cytokine expression in the whole lungs (related to** **Figure 7A** **).** The OVA-sensitized mice were challenged with vehicle alone or OVA as indicated. One day after the last challenge, their whole lungs were collected for RNA preparation. RNA pooled from 6 animals of each group was subjected to qRT-PCR to dertmine relative cytokine mRNA levels by qRT-PCR. *, *P*<0.05; ***, *P*<0.001 between OVA-challenged PLCε^+/+^ and PLCε^−/−^ mice. **Figure S3. Immunostaining for PLCε.** Paraffin-embedded sections of the lung were prepared from naïve adult PLCε^+/+^ mice and stained with the antibody against PLCε (*brown*). Nuclei were counter-stained with hematoxylin (*blue*). Representative sections containing alveolar epithelial cells (*A*), bronchial epithelial cells (*arrowhead* in *B*) and smooth muscle cells (*arrows* in *B* and *C*), are shown. *Bars,* 50 µm.
(PDF)
