# Supplementary figures and images for: Phospholipase Cε, an Effector of Ras and Rap Small GTPases, Is Required for Airway Inflammatory Response in a Mouse Model of Bronchial Asthma
Source: PLoS One. 2014 Sep 30;9(9):e108373. doi: 10.1371/journal.pone.0108373 (PMC4182471; doi:10.1371/journal.pone.0108373)

# Figure S1

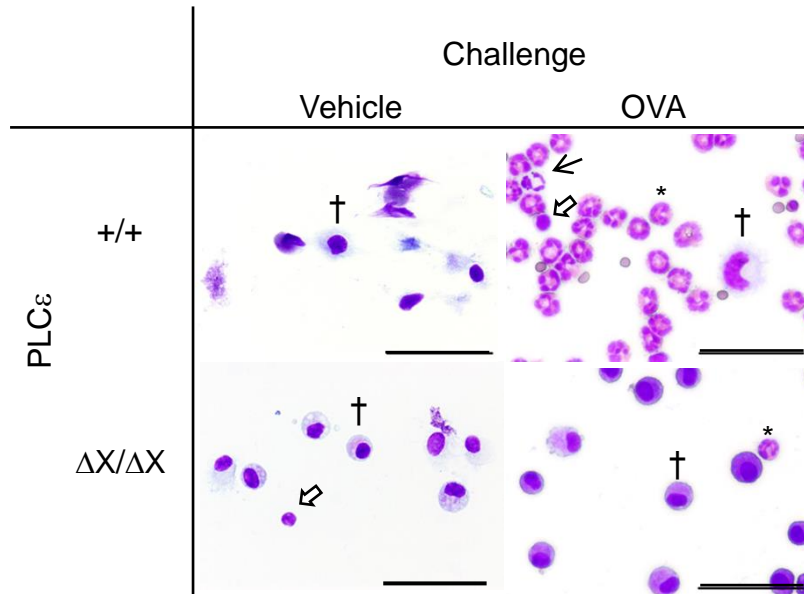

# Figure S2

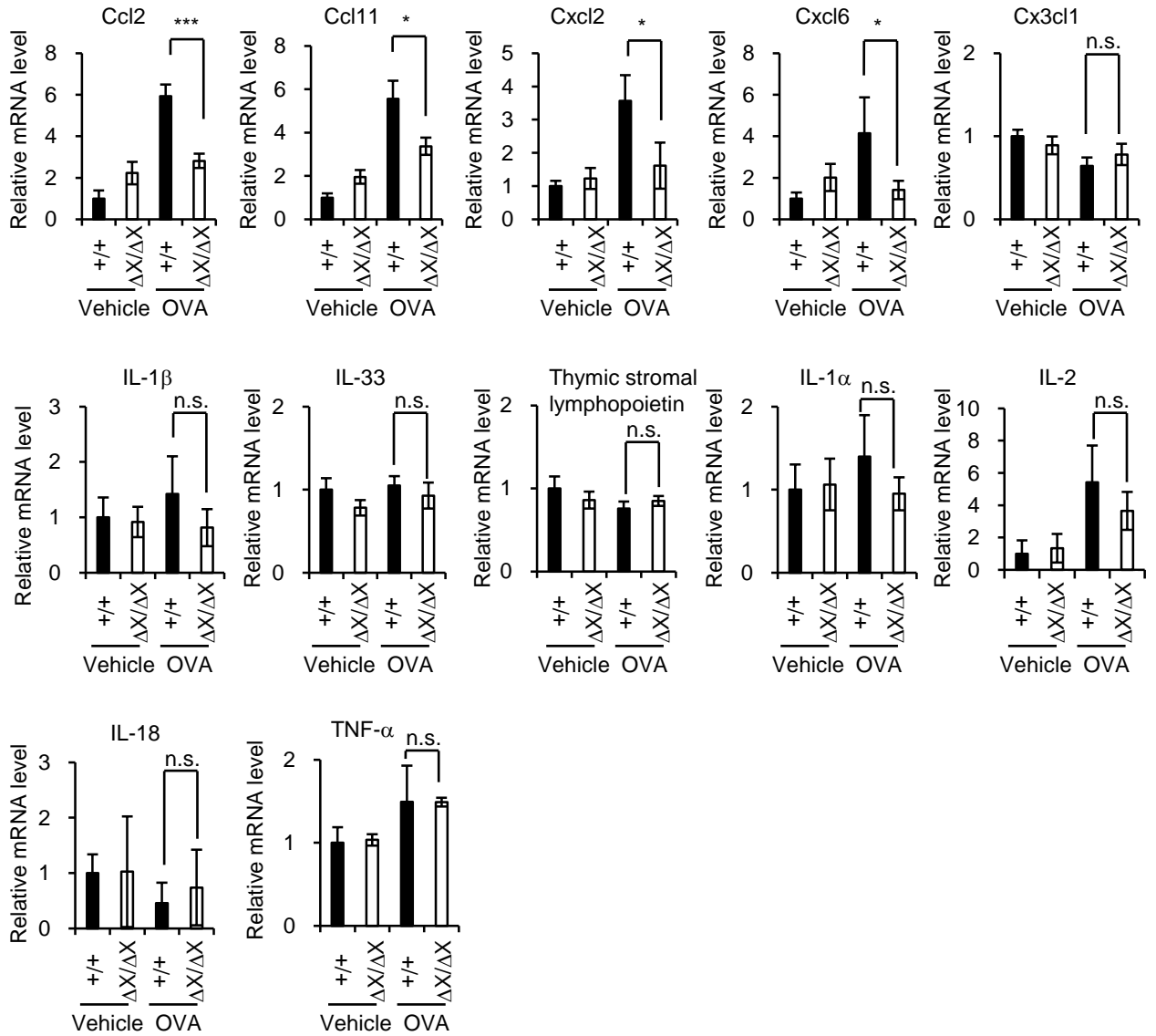

# Figure S3

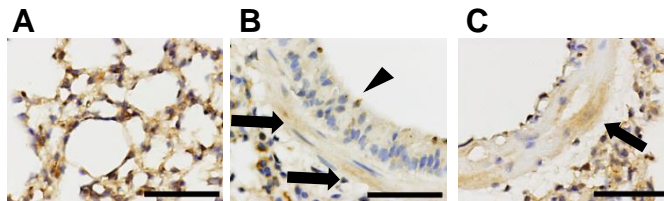

Supplement: File S1 — Figure S1. ELISA of plasma histamine levels. Plasma samples were collected from the sensitized PLCε+/+ (closed bars) and PLCε−/− (open bars) mice 24 h after the last challenge with vehicle alone or OVA. Data are expressed as the mean ± SD obtained with 3 mice of each group. Figure S2. Effects of the PLCε genotype on cytokine expression in the whole lungs (related to Figure 7A ). The OVA-sensitized mice were challenged with vehicle alone or OVA as indicated. One day after the last challenge, their whole lungs were collected for RNA preparation. RNA pooled from 6 animals of each group was subjected to qRT-PCR to dertmine relative cytokine mRNA levels by qRT-PCR. *, P<0.05; ***, P<0.001 between OVA-challenged PLCε+/+ and PLCε−/− mice. Figure S3. Immunostaining for PLCε. Paraffin-embedded sections of the lung were prepared from naïve adult PLCε+/+ mice and stained with the antibody against PLCε (brown). Nuclei were counter-stained with hematoxylin (blue). Representative sections containing alveolar epithelial cells (A), bronchial epithelial cells (arrowhead in B) and smooth muscle cells (arrows in B and C), are shown. Bars, 50 µm. (PDF) [file pone.0108373.s001.pdf]
